# Pan-cancer analysis reveals intratumoral microbial diversity in multiple cancers by amplicon technology

**DOI:** 10.3389/fcimb.2025.1549319

**Published:** 2025-09-02

**Authors:** Xudong Liu, Yuteng Yao, Aoyi Xiao, Dingyan Cao, Jingheng Zhang, Yanan Shi, Qing Zhong, Zilong He, Wenming Wu

**Affiliations:** ^1^ Laboratory Animal Research Facility, National Infrastructures for Translational Medicine, Institute of Clinical Medicine, Peking Union Medical College Hospital, Chinese Academy of Medical Science and Peking Union Medical College, Beijing, China; ^2^ The State Key Laboratory for Complex, Severe, and Rare Diseases, Peking Union Medical College Hospital, Beijing, China; ^3^ School of Life Sciences, Henan University, Kaifeng, Henan, China; ^4^ Department of General Surgery, Peking Union Medical College Hospital, Chinese Academy of Medical Science & Peking Union Medical College, Beijing, China; ^5^ Biomedical Engineering Facility of National Infrastructures for Translational Medicine, Institute of Clinical Medicine, Peking Union Medical College Hospital, Chinese Academy of Medical Sciences and Peking Union Medical College, Beijing, China; ^6^ School of Engineering Medicine, Beihang University, Beijing, China; ^7^ Beijing Advanced Innovation Center for Big Data-Based Precision Medicine, Interdisciplinary Innovation Institute of Medicine and Engineering, Beihang University, Beijing, China

**Keywords:** pan-cancer, intratumoral microbial, 16S rDNA, pseudomonas, streptococcus, prevotella

## Abstract

Despite numerous studies investigating intratumoral microorganisms and their significant roles in cancer initiation, progression, and treatment efficacy, a systematic understanding of intratumoral microorganisms remains lacking. Herein, we conducted a study using 16S rDNA data on seven types of cancer, comprising a total of 783 samples. It’s worth noting that *Pseudomonas*, *Streptococcus*, and *Prevotella* were found to be shared with the microbial communities of the seven cancers, suggesting that these may be associated with the occurrence and development of cancers. We anticipate establishing a foundation for related research and exploring potential methods for cancer treatment.

## Introduction

According to the 2024 World Health Organization report, cancer is listed in the top 10 causes of death (Global health estimates: Leading causes of death). Crosby’s study in 2022 also shows that cancer remains a major global health challenge (Crosby et al., 2022). Microorganisms in tumors were discovered over a hundred years ago (Gagliani et al., 2014). However, due to limitations in technology and potential contamination issues, studies on microorganisms within tumors have yet to be comprehensively developed. It was not until 2014 that Gagliani et al. introduced the concept of intratumoral microbiome in tumor tissue while studying bowel cancer (Gagliani et al., 2014). Subsequently, a study published in Science in 2020 conducted an in-depth analysis of intratumoral microbiomes in up to multiple of cancer tissues, revealing that the intratumoral microbes predominantly reside within tumor cells and immune cells (Nejman et al., 2020). Further studies indicate that intratumoral microbes may play an important role in the initiation and development of cancer as well as its efficacy through DNA mutations, promotion of chronic inflammation, etc (Yang et al., 2023). Therefore, the study of intratumoral microbes in cancer is necessary.

Along with the study of intratumoral microbes, some far-reaching findings have been produced in recent years. For example, Abe et al. revealed a significant positive correlation between a shorter survival time and the presence of anaerobic bacteria such as *Bacteroides*, *Lactobacillus*, and *Peptoniphilus (* Yu et al., 2025). In Chai’s microbial study of cholangiocarcinoma, *Paraburkholderia fungorumwas* was found to inhibit tumor growth through alanine, aspartate, and glutamate metabolism (Chai et al., 2023). Zhang et al. found that intra-tumoral *Fusobacterium nucleatum* further promotes the growth and immune infiltration of senescent esophageal squamous cell carcinoma cells to secretion of senescence-associated secretory phenotype, which accelerated tumor growth (Zhang et al., 2023). Li et al. pinpoint intracellular *Shewanella algae* as a foremost LM risk factor in both AI- and non-AI-treated patients (Li et al., 2024). All these results suggest a close link between intratumoral microorganisms and tumors. However, owing to the diversity of cancer types, there is still a lack of systematic understanding of intratumoral microbes.

In this study, we collected a total of 783 samples from seven cancer types, including breast, esophageal, gastric, liver, lung, pancreatic cancers and oral squamous cell carcinoma(OSCC) to study the intratumoral microbiota. In order to explore the intratumoral microbiota in detail, based on the publicly available samples, we performed various analysis strategies such as Alpha diversity index comparison, LDA analysis, and Co-abundance network analysis et al. Our goal is to provide a holistic understanding of the intratumoral microbial composition of cancer tissues through the above analyses, to advance research related to intratumoral microbiology, and to provide a scientific basis for possible therapeutic options.

## Materials and methods

### Data accession and sequence processing

For all available samples, we downloaded the pair-end sequencing files of 16S rDNA for each sample from ENA database, and all files were downloaded as FASTQ format. We performed data quality control using FastQC version 0.12.0 for the raw data of each cancer-type sample (https://www.bioinformatics.babraham.ac.uk/projects/fastqc/). For raw reads, we performed splice removal using TrimGalore version 0.6.5 with the default splice ‘AGATCGGAAGAGC’ (https://www.bioinformatics.babraham.ac.uk/projects/trim_galore/). Subsequently, the trimmed pair-end reads were merged in EasyAmplicon software using vsearch software version v2.22.1 and the merged sequences were dereplicated (Rognes et al., 2016; Liu et al., 2023). We analyzed these 16S data using uniform standards to ensure that data noise was minimized.

### Taxonomic classification and bioinformatic analysis

We denoised the dereplicated sequences to obtain ASVs using usearch software at minsize=10. Taxonomy classification was performed in EasyAmplicon using the rdp_16s_v18.fa database, and then the same types of taxa were summarized at the phylum, class, order, family, and genus levels when the raw counts were normalized to an ASV table of relative abundance groups (Liu et al., 2023). Subsequently, the visualization of the taxonomy classification of microbial communities was performed using the tax_stackplot.R script in EasyAmplicon software (Liu et al., 2023).

Using R package vegan version 2.6-8, we calculated the alpha diversity indices (Shannon index, Simpson index, observed_features index, and pielou_evenness index) of microorganisms from seven different cancer tissues (vegan: Community Ecology Package). The alpha diversity visualization was performed with R package Tidyverse version 2.0.0 (Wickham et al., 2019).The diversity of the bacterial community was assessed by the Shannon and Simpson indices, the richness of the bacterial community was assessed by the observed_features index, and the evenness of the bacterial community was assessed by the pielou_evenness index. To compare the differences in the diversity indices of microbial communities between control and case in six cancer types, we conducted an ANOVA significance test (p < 0.05). In addition, we performed β-diversity analysis based on Bray-Curtis distance using the online analysis platform www.bioincloud.tech (Gao et al., 2024). The identification of abundance-differentiated microorganisms was carried out using linear discriminant analysis (LDA) of effect sizes. LDA scores indicate the effect sizes of each ASV, and ASVs with LDA values >2.0 were defined as ASVs of differential abundance. The results obtained were used for data visualization using ggplot2 version 3.5.1 and RColorBrewer version 1.1–3 packages in R4.3.2. The online analysis platform www.bioincloud.tech was utilized to visualize the co-abundance networks of microbial communities at the genus level for each type of cancer, as well as to create a clustering heatmap of the thirteen most common bacteria, which is selected from many past researches (Eun et al; Farrell et al; Qiao et al; Tsay et al; Zhang et al; Urbaniak et al., 2016; Klann et al., 2020; Nalluri et al., 2021; Gao et al., 2024).

### Statistical analysis

We performed statistical analyses on the IBM SPSS Statistics 25 platform. We used ANOVA and LSD tests to compare the differences in each diversity index between groups. The co-abundance network among microbial taxa were calculated using Spearman rank correlation analysis. Only correlation coefficients greater than 0.4 will be showed.

## Results

### Intratumoral microbial data collection and statistics

A total of 783 samples (31.7 GB in Genome size) of seven cancer types (**Supplementary Table S1**) were selected from eight Bioprojects for this study, and 43689 ± 41918 reads (mean ± SD) were obtained from each sample after quality control (**Table 1**; **Supplementary Table S1**). There are lung cancer (29 cancerous samples; 29 healthy tissue samples), intrahepatic cholangiocarcinoma (45 cancerous samples; 49 paracancerous samples), hepatocellular carcinoma (63 cancerous samples; 61 paracancerous samples), OSCC(20 cancerous samples; 20 healthy tissue samples), breast cancer(45 cancerous samples; 23 healthy tissue samples), esophageal cancer (21 cancerous samples; 21 healthy tissue samples), gastric cancer (134 cancerous samples; 157 healthy samples), and pancreatic cancer (66 cancerous samples). Among them, we combined intrahepatic cholangiocarcinoma and hepatocellular carcinoma into liver cancer tissues for further analysis.

**Table 1:** Detailed information of seven cancer types.

Cancer types	Sample number	SRA	Bioproject
Lung	58	SRX9567656	PRJNA680529
Liver(intrahepatic cholangiocarcinoma)	94	SRX11731648	PRJNA753723
Liver (hepatocellular carcinoma)	124	SRX10336718	PRJNA714196
OSCC	40	SRX2348605	PRJNA352375
Breast	68	SRX1817289	PRJNA323995
Esophagus	42	SRX12381532	PRJNA766558
Gastric	291	SRX1992555	PRJNA310127
Pancreas	66	SRX8103970	PRJNA624822

### α diversity and β diversity of intratumoral microorganism in pan-cancer analysis

**Figure 1:**
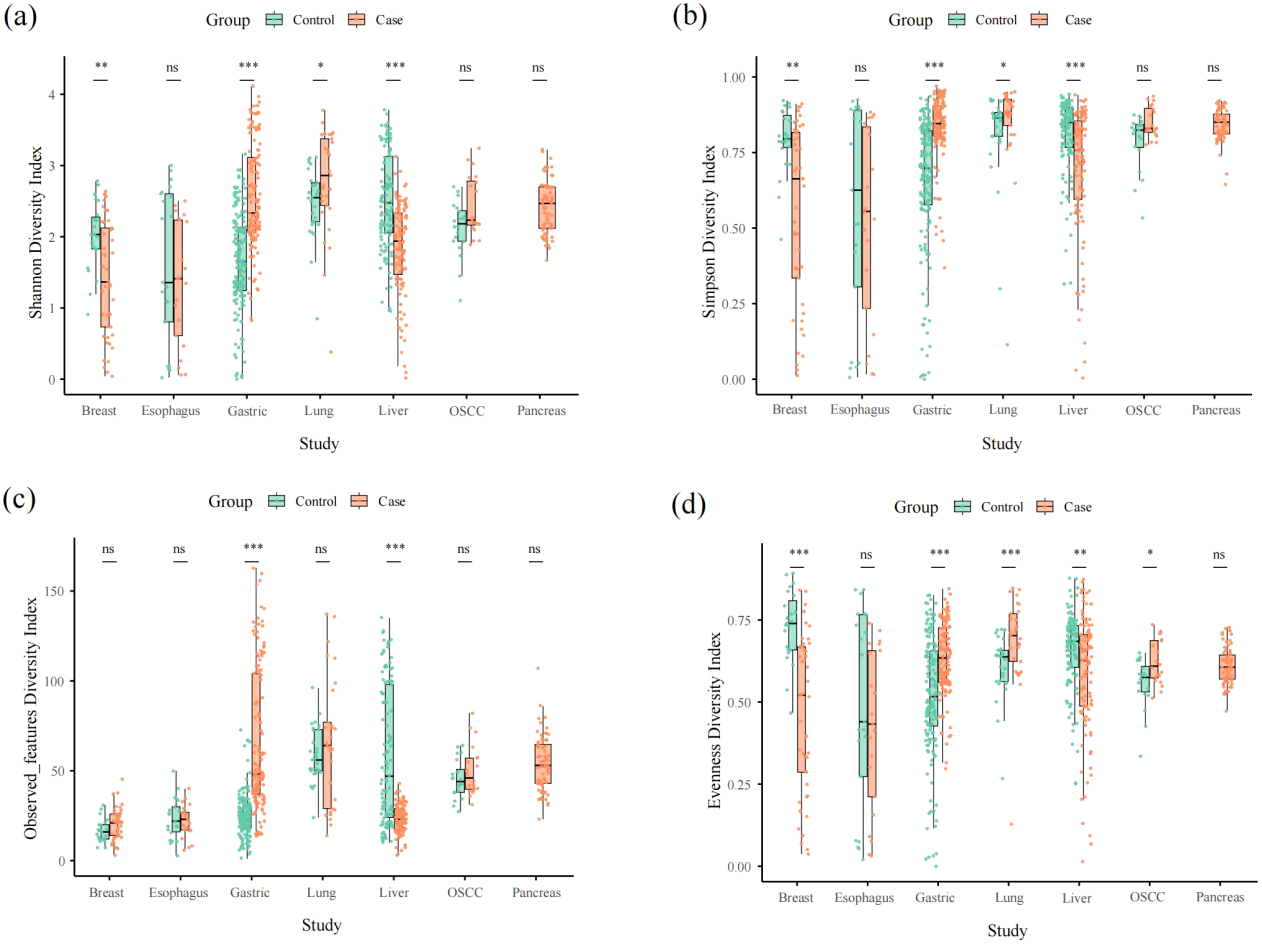
Alpha diversity box plot (Shannon, Simpson, observed_features and pielou_evenness) among seven cancer types. **(a–d)** represent shannon, simpson, observed_features and evenness aplha diversity, respectively.

In the α-diversity analysis, we found that gastric cancer and liver cancer were extremely significant difference (*p < 0.001*) in mean Shannon index and mean microbial Simpson’s index between healthy tissue samples and cancerous samples. There was no significance (*p > 0.05*) difference of mean microbial Shannon index and mean microbial Simpson’s index between healthy tissue samples and cancerous samples in esophageal cancer, OSCC and pancreatic cancer. The mean microbial Shannon index and mean microbial Simpson’s index difference in breast cancer and liver cancer were also significant difference, *p – value < 0.01* and *p – value < 0.05*, respectively (**Figures 1A, B**; **Supplementary Table S2**). The mean value of the microbial observed_features index between control and tumor of liver cancer samples and gastric cancer samples was significantly difference(*p<0.001*) (**Figure 1C**; **Supplementary Table S2**). There is no significant difference in the mean observed_features index of microbial diversity in remaining cancer types between control and case (**Figure 1C**; **Supplementary Table S2**). The mean microbial evenness index between control and case was greatly significant (*p < 001*) difference in breast tumor, gastric tumor and lung tumor (**Figure 1D**; **Supplementary Table S2**). There was significant difference in the mean microbial evenness index between control and case in liver cancer and OSCC with a different degree, *p – value < 0.01* and *p – value < 0.05*, respectively (**Figure 1D**; **Supplementary Table S2**). There was no significance difference in the mean microbial evenness index between control and case of esophageal cancer and pancreatic cancer (**Figure 1D**; **Supplementary Table S2**).

Based on the Bray-Curtis distance, principal coordinate analysis (PCoA) revealed that the similarity of microbial communities between breast cancer samples and pancreatic cancer samples is high (**Supplementary Figure S1**). A distinct trend of dispersion was observed in gastric group, suggesting a high variability of bacterial composition (**Supplementary Figure S1**).

### Taxonomy classification of intratumoral microorganism in pan-cancer analysis

**Figure 2:**
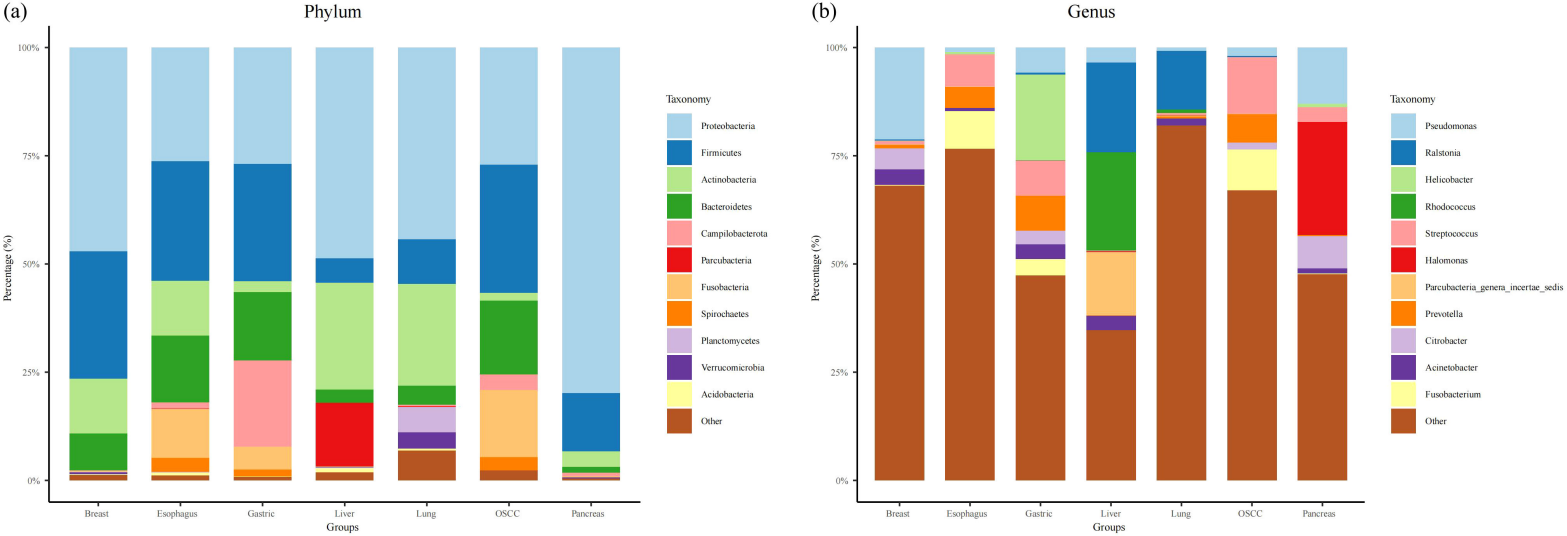
Bar plots of the phylum and genus taxonomic levels among seven cancer types. **(a, b)** represent the composition of bacteria in phylum and genus, respectively.

A total of 32 phyla, 62 classes, 109 orders, 246 families, and 721 genera were detected in Pan-cancer analysis. Overall, the intramural microorganisms of the seven different cancers were mainly composed of Proteobacteria, Firmicutes, and Actinobacteria at the phylum level, and the proportion of Proteobacteria in the seven different cancer was as follows in descending order: pancreatic cancer (79.88%), hepatocellular carcinoma (48.71%), breast cancer (47.06%), lung cancer (44.31%), OSCC (27.14%), gastric cancer (26.89%) and esophageal cancer (26.31%) (**Figure 2A**). Proteobacteria had the highest proportion in pancreatic cancer. The proportion of Firmicutes in the seven cancer samples in order of highest to lowest is as follows: OSCC (29.54%), breast cancer (29.47%), esophageal cancer (27.63%), gastric cancer (27.15%), pancreatic cancer (13.41%), lung cancer (10.35%), and liver cancer (5.60%). We found that Firmicutes had a high and approximately equal percentage in OSCC, breast, esophageal, and gastric cancer samples, and the lowest percentage in liver cancer. Actinobacteria had a high proportion of 24.74% and 23.48% in liver and lung cancer, respectively, while the lowest percentage is 1.76% in OSCC samples. Bacteroidetes was higher in OSCC, gastric, and esophageal cancer samples with 17.11%, 15.77%, and 15.41%, respectively. The lowest percentage was found in pancreatic cancer with 1.38%. Campilobacterota had the highest percentage of 19.99% in gastric cancer samples and was also present in OSCC, esophageal, lung, and breast cancer samples, but with a smaller proportion. In addition, Parcubacteria had a higher percentage of 14.69% in liver cancer. Fusobacteria and Spirochaetes had higher percentages of 15.50%, 11.39%, 5.23%, and 3.06%, 3.24%, 1.68% in OSCC, esophageal, and gastric cancer samples, respectively. Planctomycetes and Verrucomicrobia were only present and relatively high in lung cancer with 5.81% and 3.83%, respectively. In healthy tissue microbial composition, Proteobacteria, Firmicutes, Actinobacteria was also predominant (**Supplementary Figure S2**). However, the relative abundance of these microorganisms was different form tumor tissue samples (**Supplementary Figure S2**). For example, in healthy tissues of OSCC, Firmicutes had a higher relative abundance (**Supplementary Figure S2**). Compared with esophageal cancer, the relative abundance of Proteobacteria was higher in healthy esophageal tissues (**Supplementary Figure S2**).

At the genus level, the microbial composition of different cancer samples varied considerably, with *Pseudomonas* (21.26%) and *Citrobacter* (4.92%) being more prevalent in breast cancer (**Figure 2B**). In esophageal cancer, *Fusobacterium* (8.71%), *Streptococcus* (7.39%), and *Prevotella* (4.87%) accounted for the top three in descending order. In gastric cancer, the top three genera were *Helicobacter* (19.74%), *Streptococcus* (8.24%), and *Prevotella* (8.01%). *Rhodococcus* (22.69%), *Ralstonia* (20.76%), and *Parcubacteria_genera_incertae_sedis* (14.69%) had the highest percentage in hepatocellular carcinoma. *Ralstonia* (13.61%) had the highest percentage in lung cancer. In OSCC cancer, Streptococcus (13.18%), Fusobacterium (9.41%) and *Prevotella* (6.58%) had the highest proportion. Finally, in pancreatic cancer, the top three genera in descending order were dominated by *Halomonas* (26.12%), *Pseudomonas* (12.99%) and *Citrobacter* (7.53%). Also, we found that *Pseudomonas*, *Streptococcus* and *Prevotella* were present in seven cancer samples, while *Rhodococcus* and *Acinetobacter* were present in the remaining six cancers except for OSCC. *Fusobacterium* was present in all remaining cancers except liver and lung cancer. The microbial composition of healthy tissues was great difference between groups (**Supplementary Figure S2**). It was evident that *pseudomonas* which had the most relative abundance in tumor tissues was less prevalent in healthy tissues (**Supplementary Figure S2**).

### Identification of abundance differential intratumoral microorganisms in pan-cancer analysis

**Figure 3:**
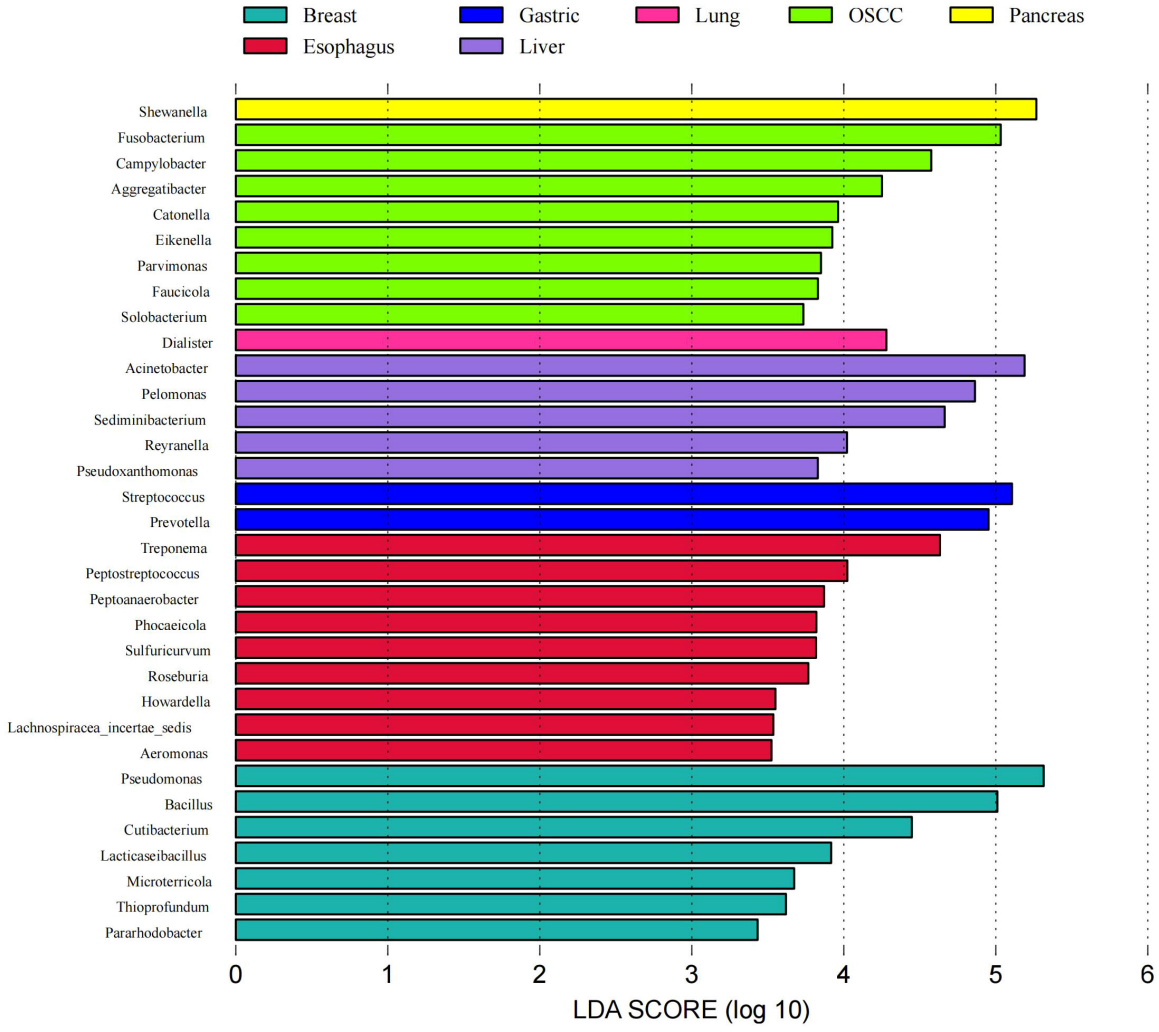
LDA analysis implied differentially abundant microorganisms among seven cancer types (LDA Score > 2).

We performed abundance differential microorganisms analysis by Lefse (LDA>2). There were 13, 19, 10, 2, 3, 19 significant abundance differential microorganisms in tumor group from breast cancer, esophageal cancer, gastric cancer, liver cancer, lung cancer and OSCC, respectively (**Supplementary Figure S3**). Then, the number of microorganisms with significant differences among different cancers, from high to low, was esophageal cancer (9), OSCC (8), breast cancer (7), hepatocellular carcinoma (5), gastric cancer (2), lung cancer (1), and pancreatic cancer (2) (**Figure 3**). *Pseudomonas* (LDA>5), *Bacillus* (LDA>4), *Cutibacterium* (LDA>4), etc. were significantly enriched in breast cancer *Treponema* (LDA>4), *Peptostreptococcus* (LDA>4), *Peptoanaerobacter* (LDA>3), etc. were enriched in esophageal cancer. *Streptococcus* (LDA>5), *Prevotella* (LDA>4) were significantly enriched in gastric cancer. *Acinetobacter* (LDA>5), *Pelomonas* (LDA>4), *Sediminibacterium* (LDA>4), etc. were significantly enriched in liver cancer. *Fusobacterium* (LDA>5), *Campylobacter* (LDA>4), *Aggregatibacter* (LDA>4), etc. were significantly enriched in OSCC. *Dialister* (LDA>4) and *Shewanella* (LDA > 5) were significantly enriched in lung cancer and pancreatic cancer, respectively.

### Co-abundance networks of intratumoral microorganisms in pan-cancer analysis

**Figure 4:**
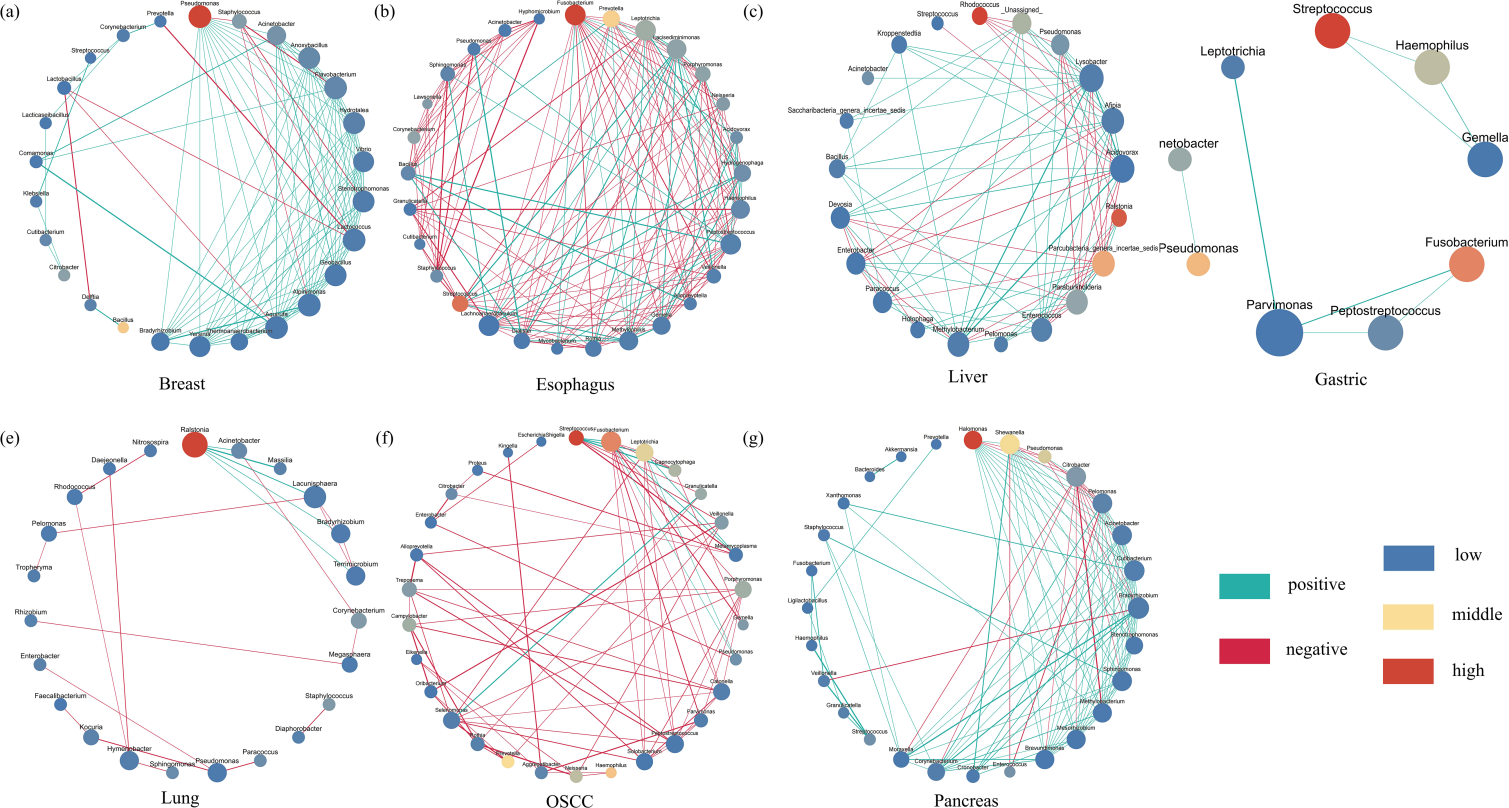
The Co–abundance network of six cancer types. The blue color represents least abundant, yellow color represents intermediate abundance and red represents the most abundant. The light green color of connecting lines represents negative correlation and red represents positive correlation. The size of the nodes indicates degree number and set from 30 to 60. The biggest one means that it has most degrees, and the smallest one means least degrees. Only correlation coefficients greater than 0.4 will be showed. The correlation coefficients p – value threshold is 0.05. **(a–g)** represent the co-abundance network of bacteria in breast, esophagus, liver, gastric, lung, oscc and pancreas, respectively.

We performed Co-abundance networks analysis of seven microorganisms with different cancer types (correlation coefficient >0.4, p-value<0.05) (**Figure 4**). We found that *Pseudomonas* (relative abundance =20.82, degree=14), *Fusobacterium* (relative abundance =8.56, degree=17), *Streptococcus* (relative abundance =8.53, degree=2), and *Ralstonia* (relative abundance =13.43,degree=5) all had high abundance and high degrees, which were predominant in breast, esophageal, gastric, and lung cancers, respectively (**Figures 4A,B,D,E**; **Supplementary Tables S3**, **S4**). In addition, in breast cancer network, there was a positive correlation (correlation index >0.6) between *Pseudomonas* and *Staphylococcus* significantly enriched bacteria(LDA > 4). In esophageal cancer network, there was a negative correlation (correlation index< -0.6) between *Fusobacterium* and significantly enriched genera (*Peptostreptococcus*, LDA>3). There were more connections among esophageal cancer network in genera. In liver cancer network, *Ralstonia* (relative abundance =20.66,degree=5), *Rhodococcus*(relative abundance = 22.69, degree = 5) and *Lysobacter* (relative abundance =1.19,degree=15) played an important role, with first two having the higher abundance and lower degree and the latter had lower abundance and highest degree (**Figure 4C**). In the OSCC network, *Streptococcus* (relative abundance =13.22, degree=6) had the highest abundance and medium degree and *Fusobacterium* (relative abundance =9.56, degree=11) had the highest degree and lower abundance (**Figure 4F**). *Leptotrichia* (relative abundance =6.02, degree=9) had higher abundance, all three played a key role in OSCC microbiological network. Among pancreatic and esophageal cancer microorganisms, *Halomonas* and *Shewanella* played key roles, both of which had higher abundance and higher degrees (**Figure 4G**). We found mostly positive correlations in breast, gastric, liver, and pancreatic cancers, whereas there was no distinct patterns of correlation among esophagus, lung, and OSCC microbiological network.

### The common intratumoral microorganisms profiling in pan-pancer analysis

**Figure 5:**
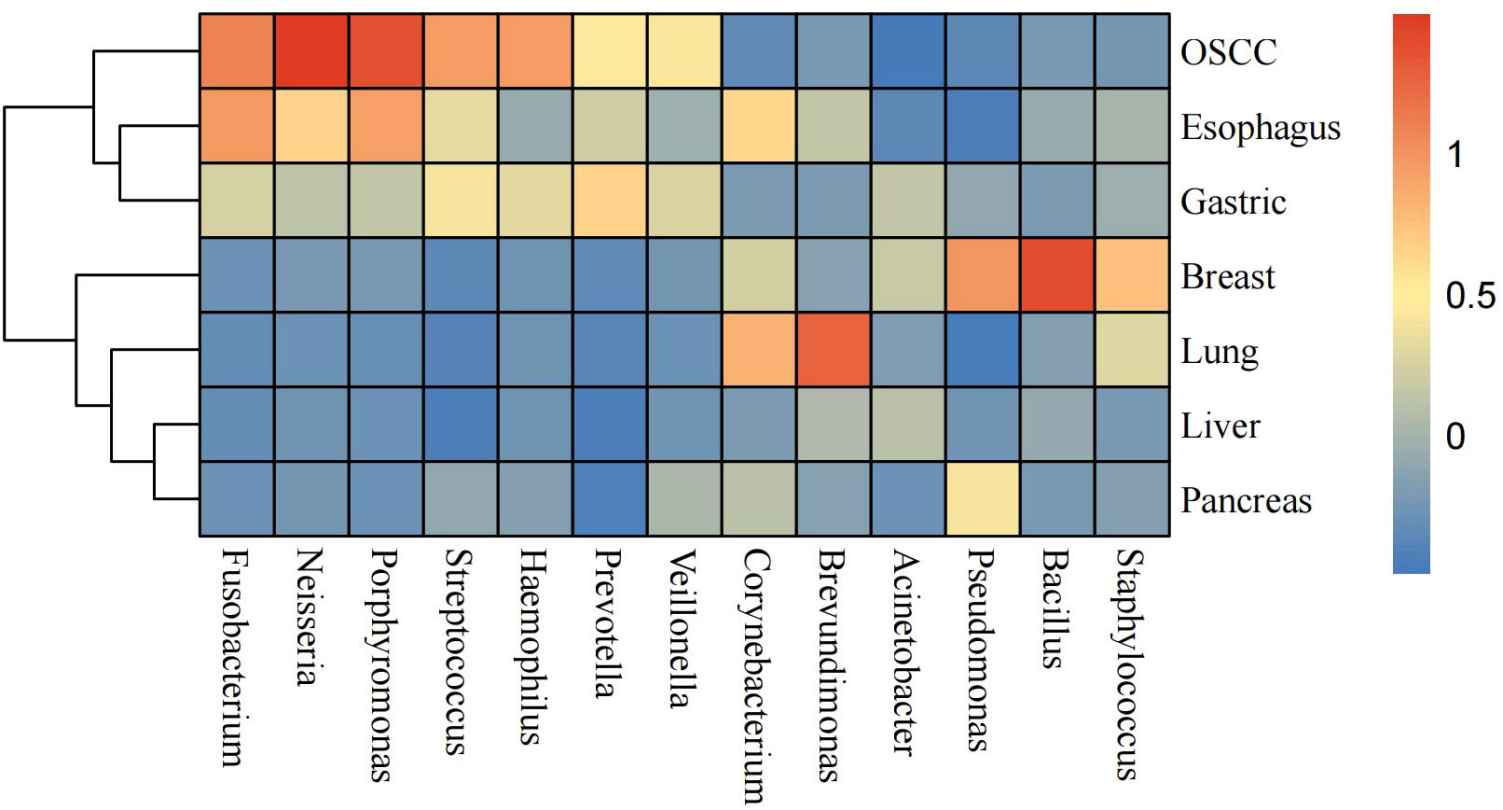
Heatmap of 13 selected most common intratumoral microorganisms at the genus level. The blue color represents less abundant, yellow color represents intermediate abundance and red represents the most abundant.

We investigated 13 common intratumoral microorganisms (genus level) abundance profiling of seven cancers. The abundance of the thirteen microorganisms varied widely among different cancers (**Figure 5**). We found that the *Neisseria*, *Porphyromonas*, and *Fusobacterium* exhibited higher abundances in OSCC samples. In esophageal cancer samples, *Fusobacterium*, *Porphyromonas*, and *Corynebacterium* were found to be more abundant. In gastric cancer tissues, *Prevotella* and *Streptococcus* showed higher abundances, while in breast cancer tissues, *Bacillus*, *Pseudomonas*, and *Staphylococcus* were more enriched. *Brevundimonas* and *Corynebacterium* were found to have higher abundances in lung cancer. Notably, there were no high-abundance genera identified in liver and pancreatic cancer samples. We found the types and abundance of common bacteria were relatively high in OSCC samples. In summary, the microbial profiling pattern of esophageal and gastric cancer samples are relatively similar, as so as liver and pancreatic cancer samples.

## Discussion

Currently, intratumoral microbiology research is hot but controversial. Nejman et al. conducted the first more comprehensive and systematic study of intratumoral microbiology in 2020 (Nejman et al., 2020). This has sparked a boom in intratumoral microbial research. In contrast to the study by Nejman et al., we analyze five different cancer types to provide a more comprehensive situation for more cancer types. Similarly, we all collect a large number of samples to diminish the error made by samples. Uniquely, we offer refined co-abundance network for each cancer type (Nejman et al., 2020). Sepich-Poore et al. in 2021 explored intratumoral microbes in cancer therapy by discovering that intratumoral bacteria produce a new tumor antigen (Sepich–Poore et al., 2021). Qu et al. in 2022 explored the microbial composition of hepatocellular carcinoma tissues and identified new diagnostic markers for primary liver tumors (Qu et al., 2022). Research articles on intratumoral microbes have been climbing in recent years. At the same time, some have raised concerns; for example, Ge et al. inferred in 2024 that the presence of microorganisms within tumors is a result of technical errors (Ge et al., 2024). In our study, we utilized the concept of pan-cancer to conduct a meta-analysis of intratumoral microorganisms across 783 samples of 16S rDNA from up to seven different cancer types, thereby maximizing the potential evidence for the presence of intratumoral microorganisms.

In our result, we found gastric cancer and liver cancer were all significant difference (p < 0.001) of the four diversity indices except evenness diversity index of liver cancer (p < 0.001). Noticeably, there was a great difference. The mean value of four diversity indices in case were significant higher than control in gastric cancer and it was opposite in liver cancer. In taxonomy classification at the phylum level, we found that the top three phyla in OSCC cancer were Firmicutes, Proteobacteria, and Bacteroidetes, respectively. In different OSCC studies, it coincided with Su’s findings (Su et al., 2021). In taxonomy classification at the genus level, the most prevalent genus in our study was *Streptococcus*, which is consistent with Su’s research (Su et al., 2021). Furthermore, in our study, *Fusobacterium* and *Prevotella* exhibited higher relative abundances, a conclusion that aligns with the findings of Michikawa et al. (2022). As a bridge between different colonizers in oral cavity, *Fusobacterium* will increase uncertainty of biofilms with an abnormal abundance (Ye et al., 2024). In breast cancer tissues, we found that Proteobacteria, Firmicutes, Actinobacteria, and Bacteroidetes were the most abundant phyla. This finding is consistent with the results of Klann et al. in various breast cancer studies (Klann et al., 2020), but it differs from the results of Urbaniak et al. (2014), where Firmicutes was the most abundant phylum (Urbaniak et al., 2014). At the genus level, *Pseudomonas* was overwhelmingly dominant. However, Urbaniak et al. (2016) found that *Staphylococcus*, *Enterobacteriaceae*, and *Bacillus* were more highly represented in breast cancer (Urbaniak et al., 2016). Meanwhile, German et al. found that *Ralstonia* and *Staphylococcus* were also higher in breast cancer (German et al., 2023). There is a big difference between our study and two other different studies. This may be related to the fact that the microbial composition of breast cancer may vary with stages (Smith et al., 2019). The abundance of *Fusobacterium*, which had been proved to worsen cancer in breast through lectin Fap2, is low (Parhi et al., 2020). Regarding the microbial composition in esophageal cancer, we found that in various esophageal cancer studies, both at the phylum and genus levels, our results were similar to those of Liu et al. and Wang et al (Liu et al., 2018; Wang et al., 2021). The only difference is that Campylobacterota exhibited a relatively higher abundance in our study. In gastric cancer, we found that compared to other gastric cancer studies, Campylobacterota exhibited a higher abundance at the phylum level in our research compared to Shao’s study, which aligns with its characteristic extensive presence in the digestive system (Shao et al., 2019). Further, Liu et al. found the infiltration level of Tregs was negatively correlated with microbial abundance, which indicates that the higher abundance of gastric tumor microbiome may pose an adverse impact on therapy (Liu et al). The higher abundance of *Fusobacterium* may induce organelle dysfunction by up-regulating the expression of related genes and promote cancer metastasis (Zhou et al., 2022). In various studies of microbial composition in liver cancer, we observed that our results were similar to those of Qu et al. at the phylum level (Qu et al., 2022). However, there were significant differences at the genus level. *Acinetobacter* was the dominant genus in Qu’s research, whereas it constituted a relatively lower proportion in our study (Qu et al., 2022). Maybe it is not the sequencing depth effects, because we normalized the raw data and took relative abundance to analysis. Subsequent plans are to validate the distribution of this microbiota in liver cancer by RNA single-cell sequencing, microbial single-cell sequencing, and microbial culture. What’s more, Xue et al. discovered the metabolism influences made by liver tumor microbiome, presenting that the metabolites of 13Z, 16Z-docosadienoic acid is overexpressed in liver tumor (Xue et al., 2024). In the microbial composition of lung cancer, our study identified Proteobacteria as the dominant phylum, in contrast to Lee’s research, where Bacteroidetes was the prevalent phylum and represented a smaller proportion in our findings (Lee et al., 2016). Additionally, *Ralstonia* showed a higher relative abundance in our study compared to its lower proportion in Lee’s research (Lee et al., 2016). Regarding the microbial composition in pancreatic cancer, we found that our results were highly similar to those of Nejman et al. at the phylum level across different studies (Nejman et al., 2020). The high abundance of Proteobacteria will lead to T cell anergy in a Toll-like receptor-dependent manner, accelerating tumor progression (Pushalkar et al., 2018). Noticeably, the presence of *Fusobacterium* will mediate tumorigenesis and metastasis by promoting the synthesis of GMCSF and CXCL1 in pancreas (Nejman et al., 2020). Interestingly, Josie et al. innovated Electro-antibacterial therapy to enhance intracellular bacteria clearance in pancreatic cancer cells, which is a potential method to cure pancreatic cancer (Duncan et al., 2024). It is noteworthy that we detected the presence of *Pseudomonas*, *Streptococcus* and *Prevotella* in the microbial compositions of the seven cancer types we studied, suggesting a potential close association between these genera and cancer.

From our results, we observed that there are significant abundant differential microorganisms in cancers such as breast cancer, esophageal cancer and OSCC, while pancreatic cancer and lung cancer exhibited fewer abundant differential microorganisms. Additionally, in the comparison between the microbial composition of normal tissue and cancer tissue, we found that *Treponema* was significantly enriched in three types of cancer tissue, which indicated that it was likely consistent with cancer.

Based on the existing researches of intratumoral microorganisms, we selected thirteen genera that are frequently found in various cancer tissues (according to Xue et al.) (Xue et al., 2023; Xuan et al., 2024). Of these thirteen genera, most are parthenogenetic anaerobes or anaerobes, and only *Neisseria* is aerobic. We can see that *Neisseria* is only found in OSCC, esophageal cancer, and gastric cancer, which may be related to human physiology. *Fusobacterium* is an anaerobic bacterium commonly associated with various infections and inflammations. We found its presence in OSCC, esophageal cancer, and gastric cancer, suggesting that this genus may possess potential pathogenicity. Additionally, due to its reported association with multiple cancers, it is also referred to as “oncobacterium” (Brennan and Garrett, 2019; McIlvanna et al., 2021; Alon–Maimon et al., 2022). According to the results, *Brevundimonas* was found to be abundantly present in lung cancer, while it was almost absent in other cancers. Furthermore, this bacterium is often associated with the occurrence of pneumonia, indicating that it may have a greater tendency to inhabit lung tissue and exhibit pathogenic potential (Hassan et al., 2023). We found *Bacillus* and *Staphylococcus* to be present in large numbers in breast cancers. Noticeably, Liu et al. found that there are positive correlations between tumoral Staphylococcus and CD8+ TIL activity exclusively in triple-negative breast cancer, demonstrating the effect of Staphylococcus on immunity (Liu et al., 2024). In general, *Bacillus* is only present in the environment and is not pathogenic, but certain species such as *Bacillus anthracis* can sometimes cause serious illness (Atlas, 1998; Watson et al., 2017). Since our samples could only be localized to the genus level, we were unable to determine which species of *Bacillus* were enriched in breast cancer. Finally, we found that these thirteen bacteria had low abundance or were absent in liver cancer and pancreatic cancer, suggesting that the microbial communities in these cancers may be dominated by other specific taxa.

However, there are some shortcomings in our study. Firstly, our study is based on online public data and 16S rDNA data, which cannot be precisely localized to the species level. Secondly, this study lacks the validation of wet experiments, which will be added in the future. We have provide a more comprehensive understanding of intratumoral microbiome. However, We hope to further investigate the composition of intratumoral microorganisms systematically through more pan-cancer samples, which will lay the foundation for related research and provide a scientific basis for possible cancer treatment options. In the future, more researches on microbiome metabolomics may further elucidate the influences of microbiome posed to tumor.

## Conclusion

In summary, we conducted a more systematic study of intratumoral microbial communities in seven different cancer types based on 16S data in pan-cancer analysis. The microbial communities of different cancers were found to have differences but also commonalities in diversity and species composition. Four genera, *Pseudomonas*, *Streptococcus*, and *Prevotella* were found to be common to the microbial communities of the seven cancers, suggesting that these microorganisms may be associated with the occurrence and development of cancer. In addition, it is noteworthy that we identified the core microorganisms in the intratumoral microbial interactions network of different cancers, which may help us to study the tumor microenvironment in depth. We hope that our results will lay the foundation for further research in the field of intratumoral microbes and provide new ideas for cancer diagnosis and treatment.

## Data Availability

The original contributions presented in the study are included in the article/**Supplementary Material**. Further inquiries can be directed to the corresponding authors.
