# Supplementary figures and images for: Pan-cancer analysis reveals intratumoral microbial diversity in multiple cancers by amplicon technology
Source: Front Cell Infect Microbiol. 2025 Sep 2;15:1549319. doi: 10.3389/fcimb.2025.1549319 (PMC12438974; doi:10.3389/fcimb.2025.1549319)

PERMANOVA:  $R^2=0.42$ ,  $F=46.24$ ,  $P=0.001$

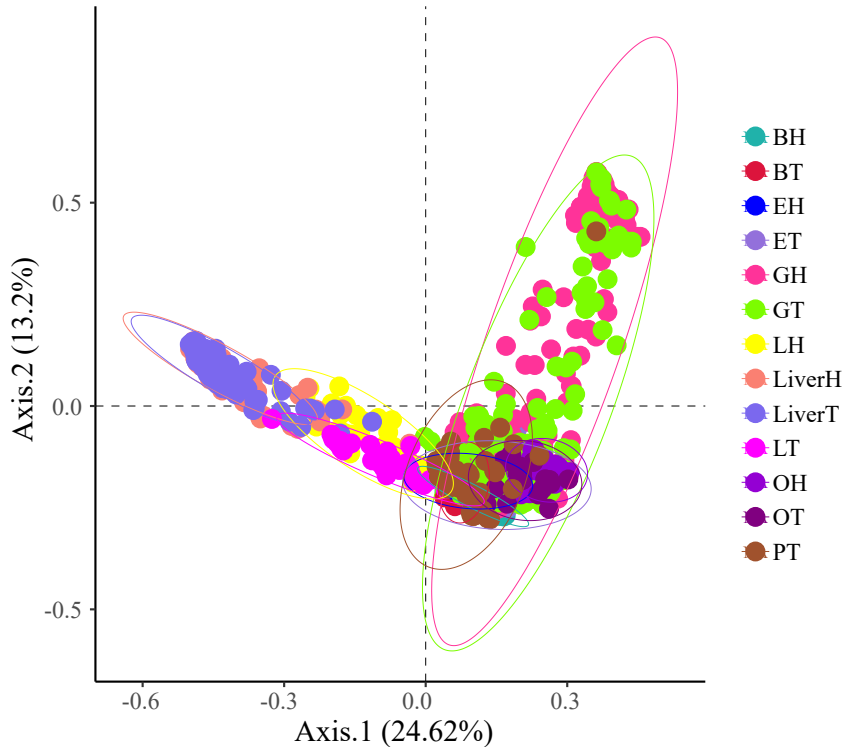

Supplement: Supplementary file 5 [file DataSheet1.pdf]

(a)

## Phylum

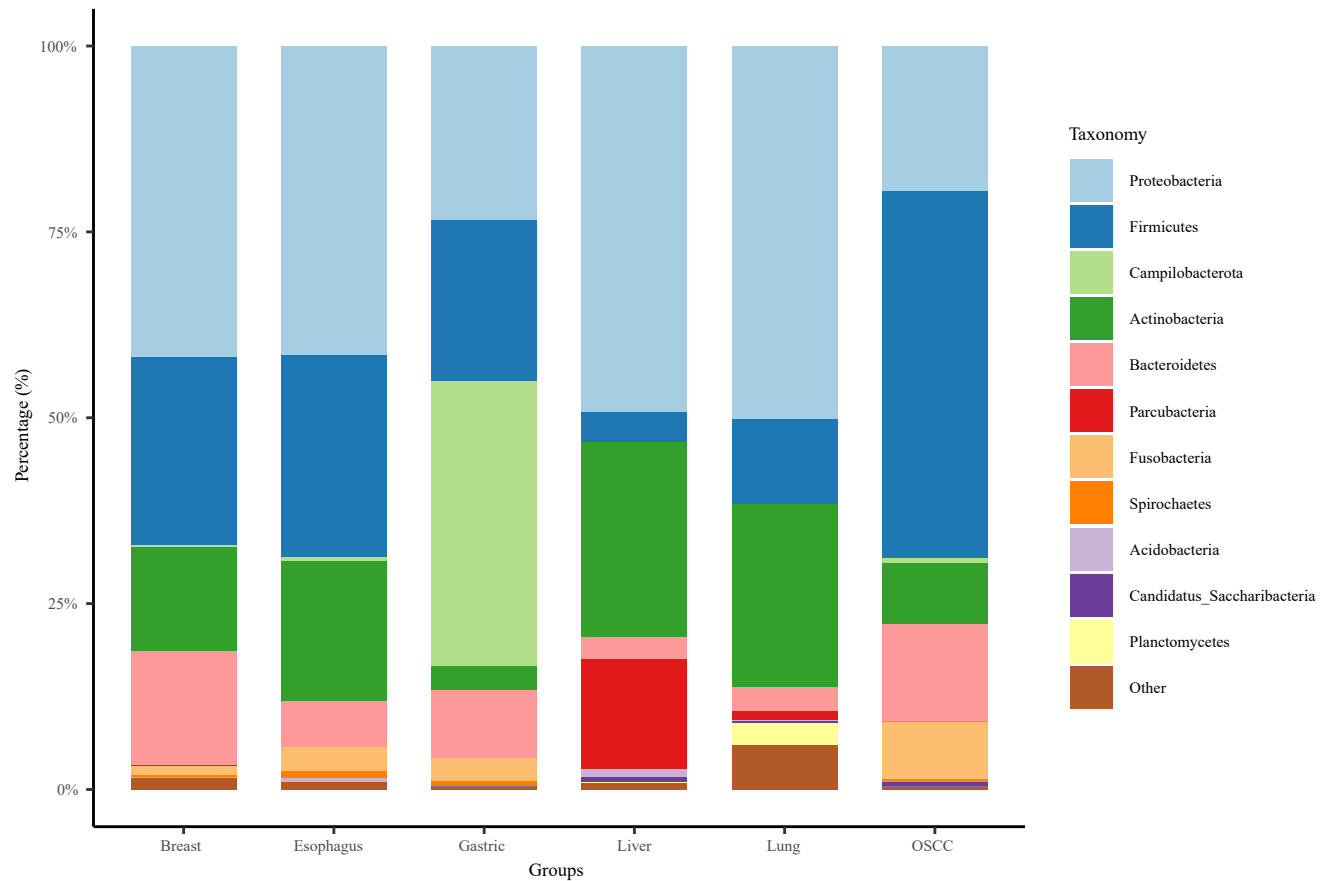

(b)

## Genus

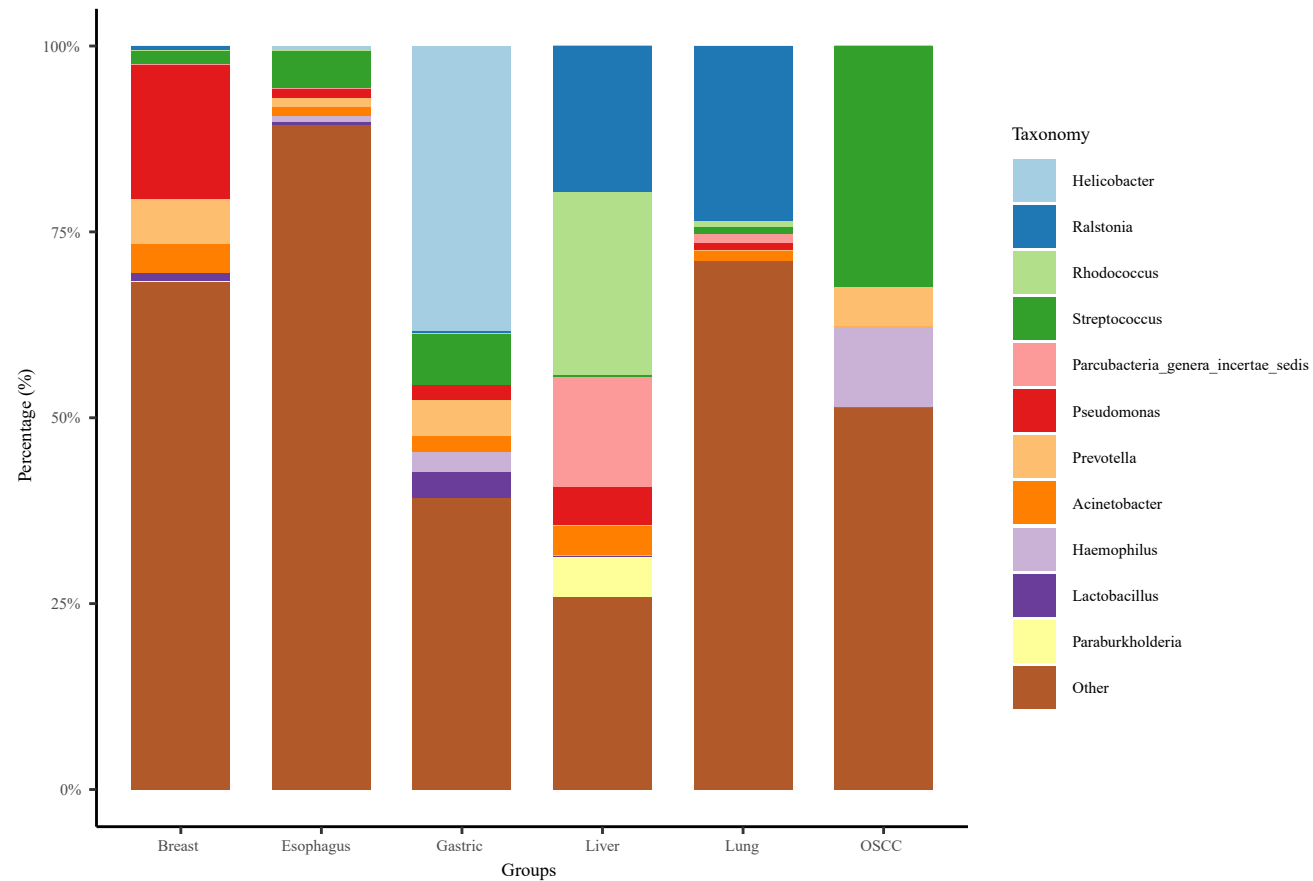

Supplement: Supplementary file 6 [file DataSheet2.pdf]

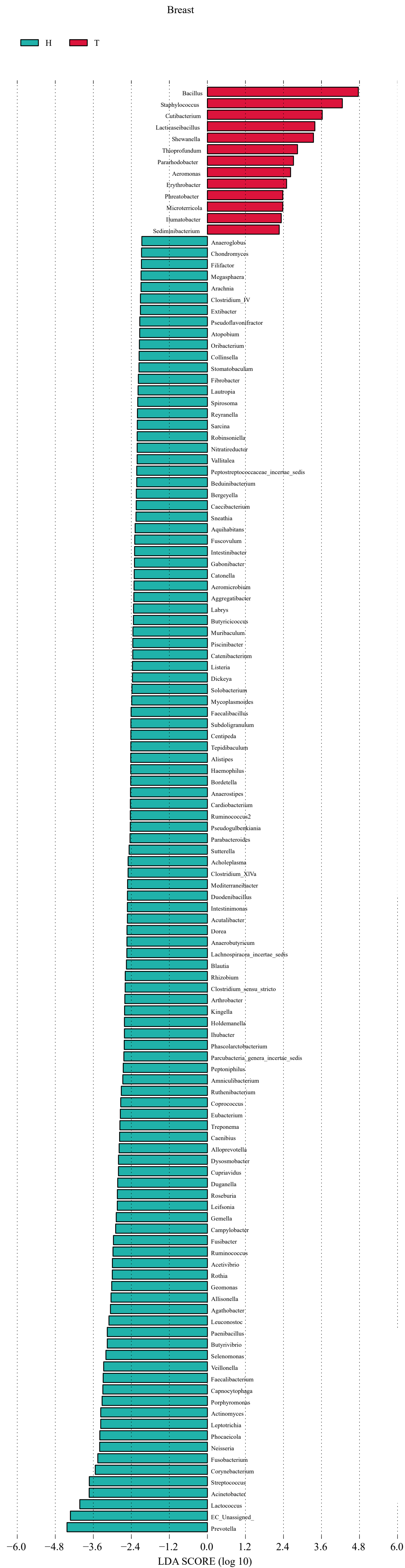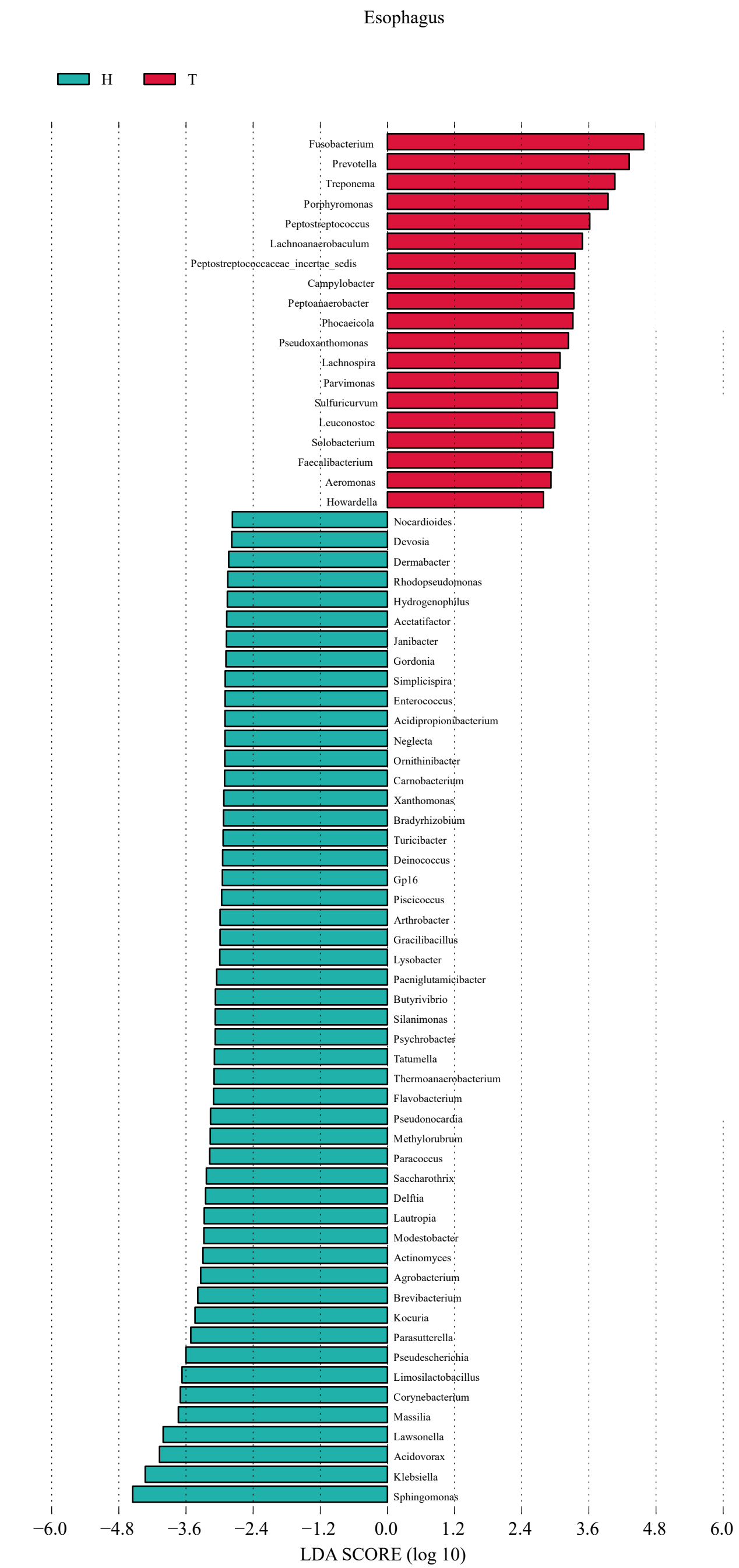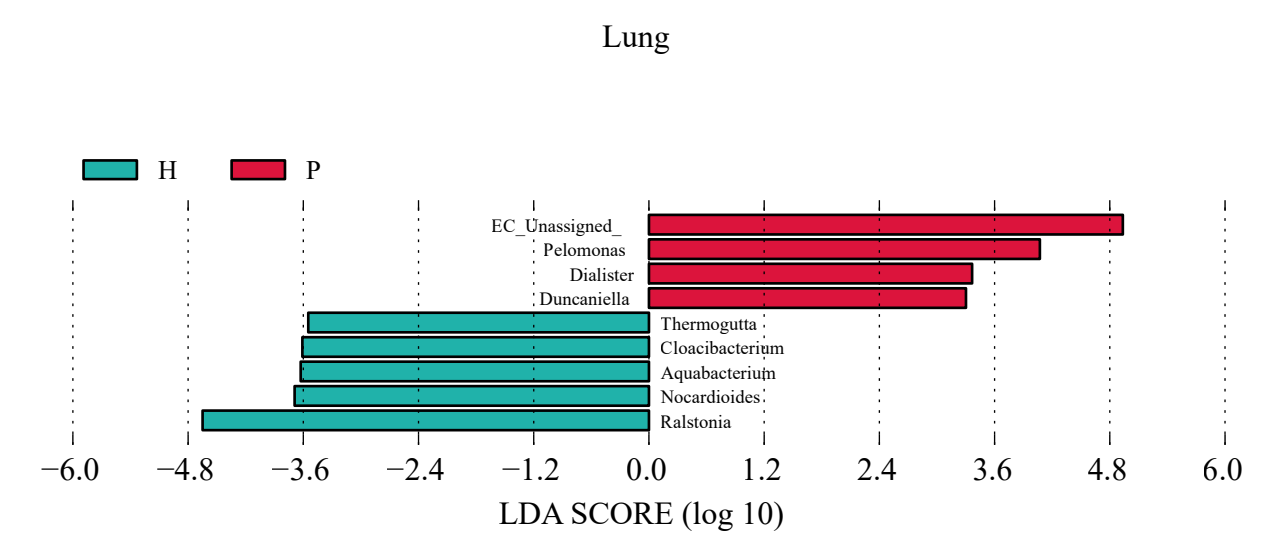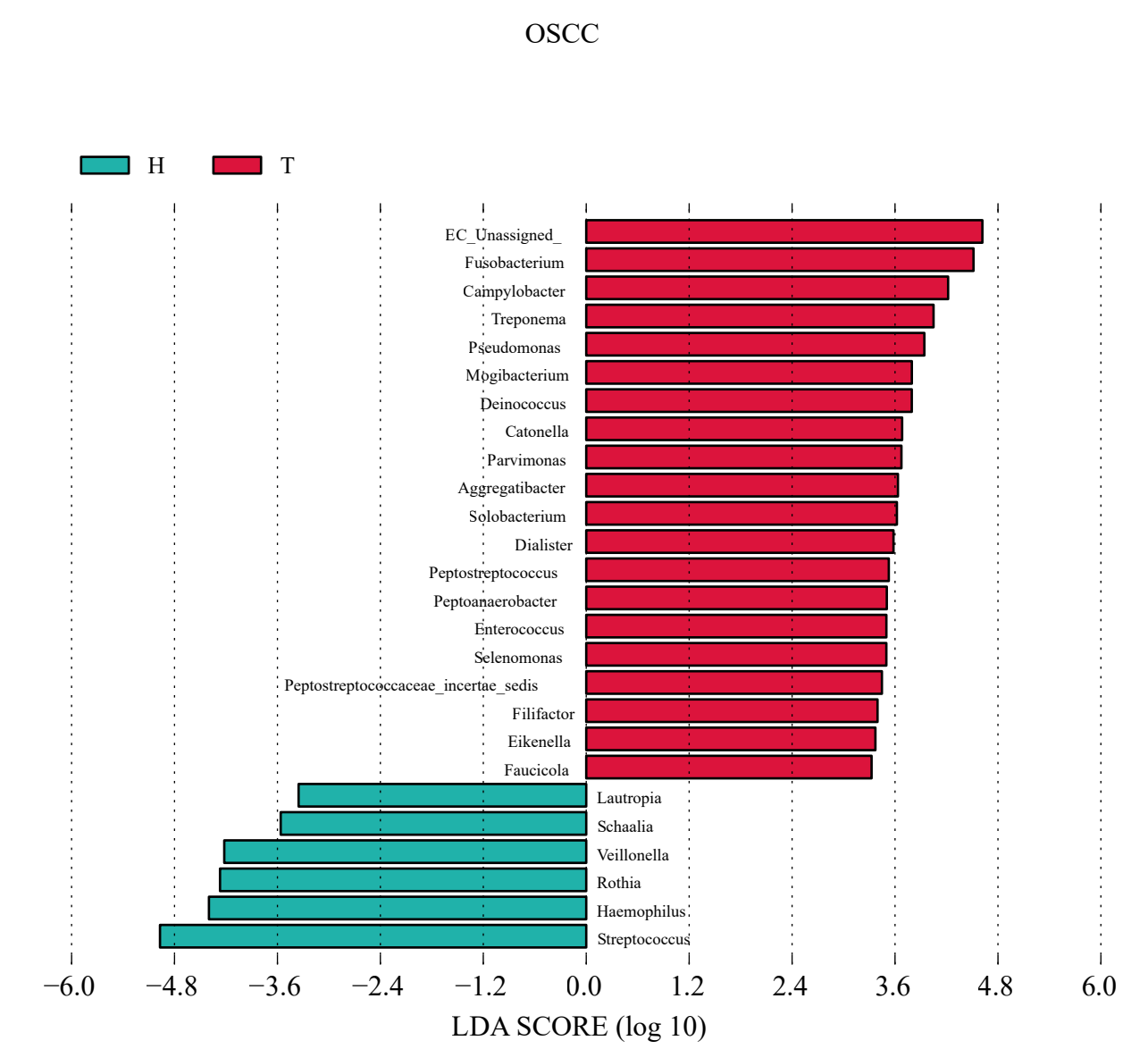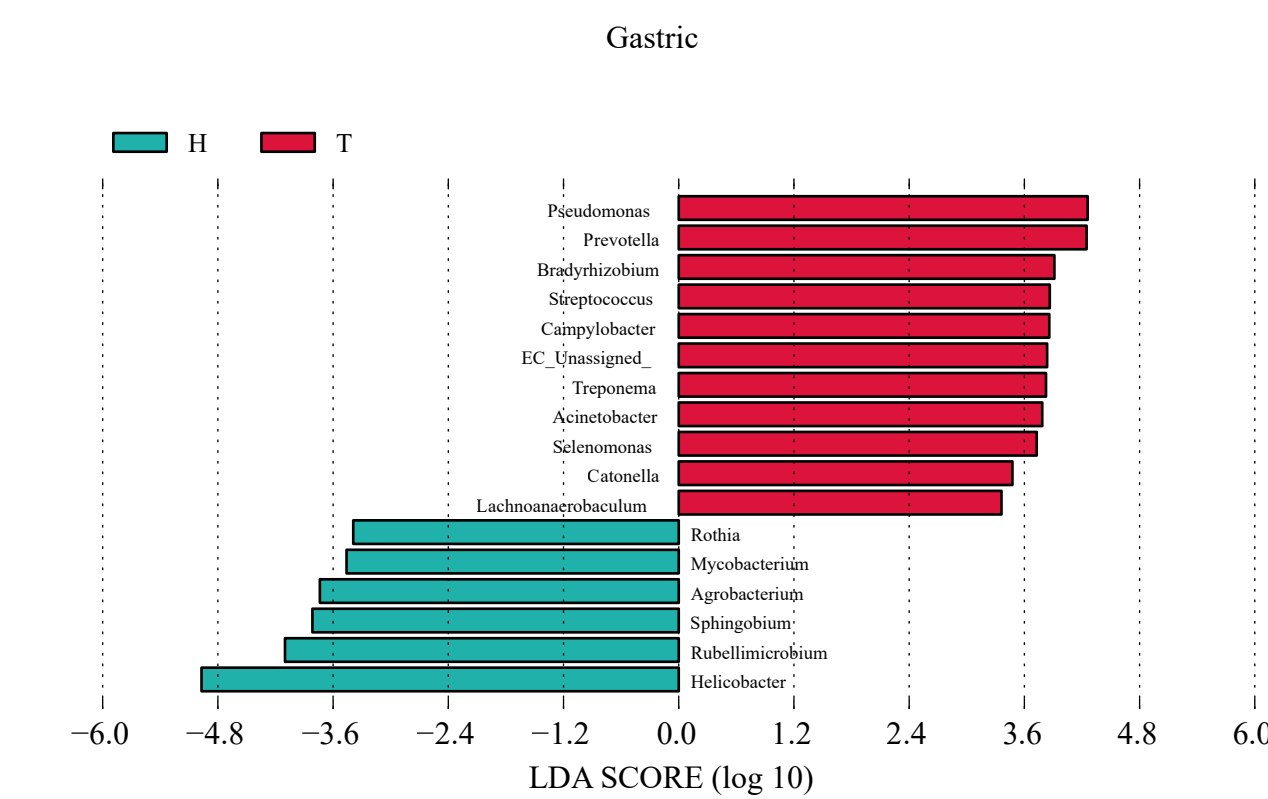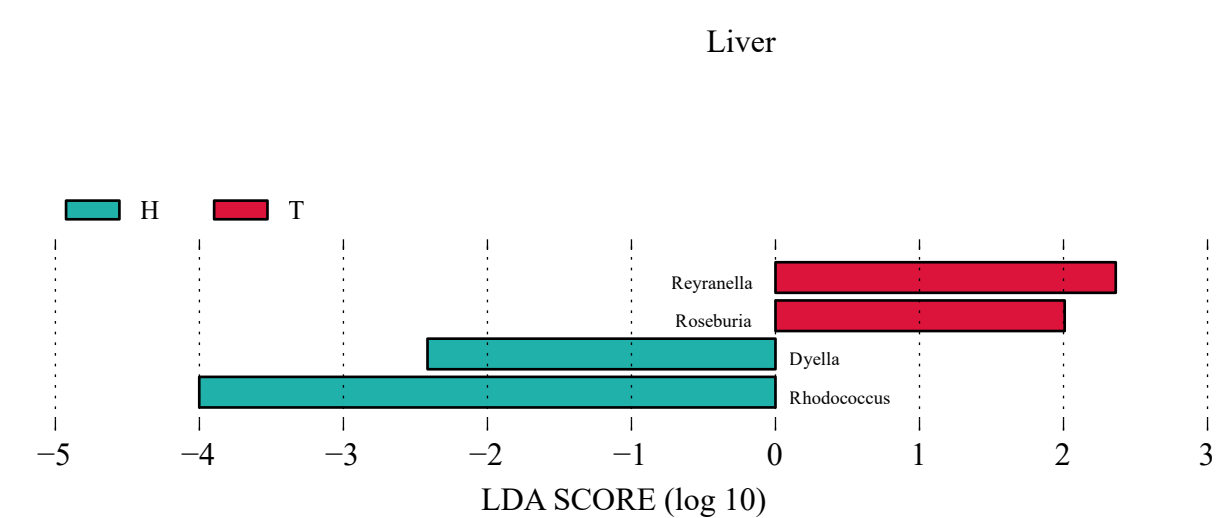

Supplement: Supplementary file 7 [file DataSheet3.pdf]
